# Supplementary material for: Circ-CREBBP inhibits sperm apoptosis via the PI3K-Akt signaling pathway by sponging miR-10384 and miR-143-3p
Source: Commun Biol. 2022 Dec 7;5:1339. doi: 10.1038/s42003-022-04263-2 (PMC9729231; doi:10.1038/s42003-022-04263-2)
Supplement: Supplementary file 2 — Supplementary Information [file 42003_2022_4263_MOESM2_ESM.pdf]

a

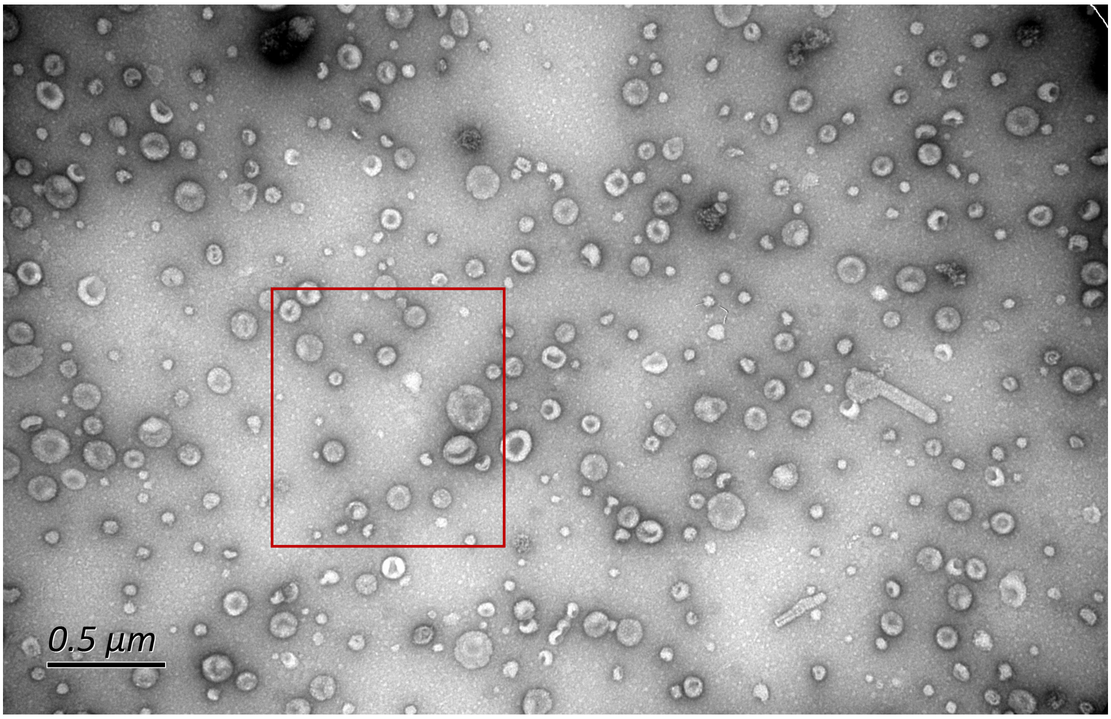

b

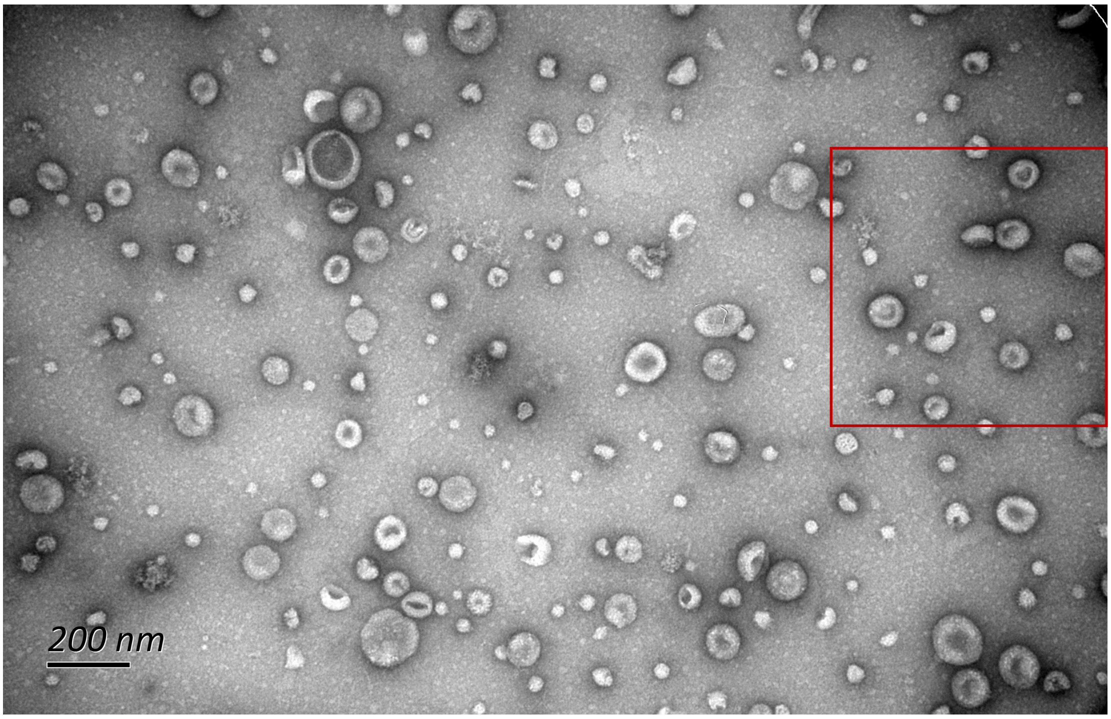

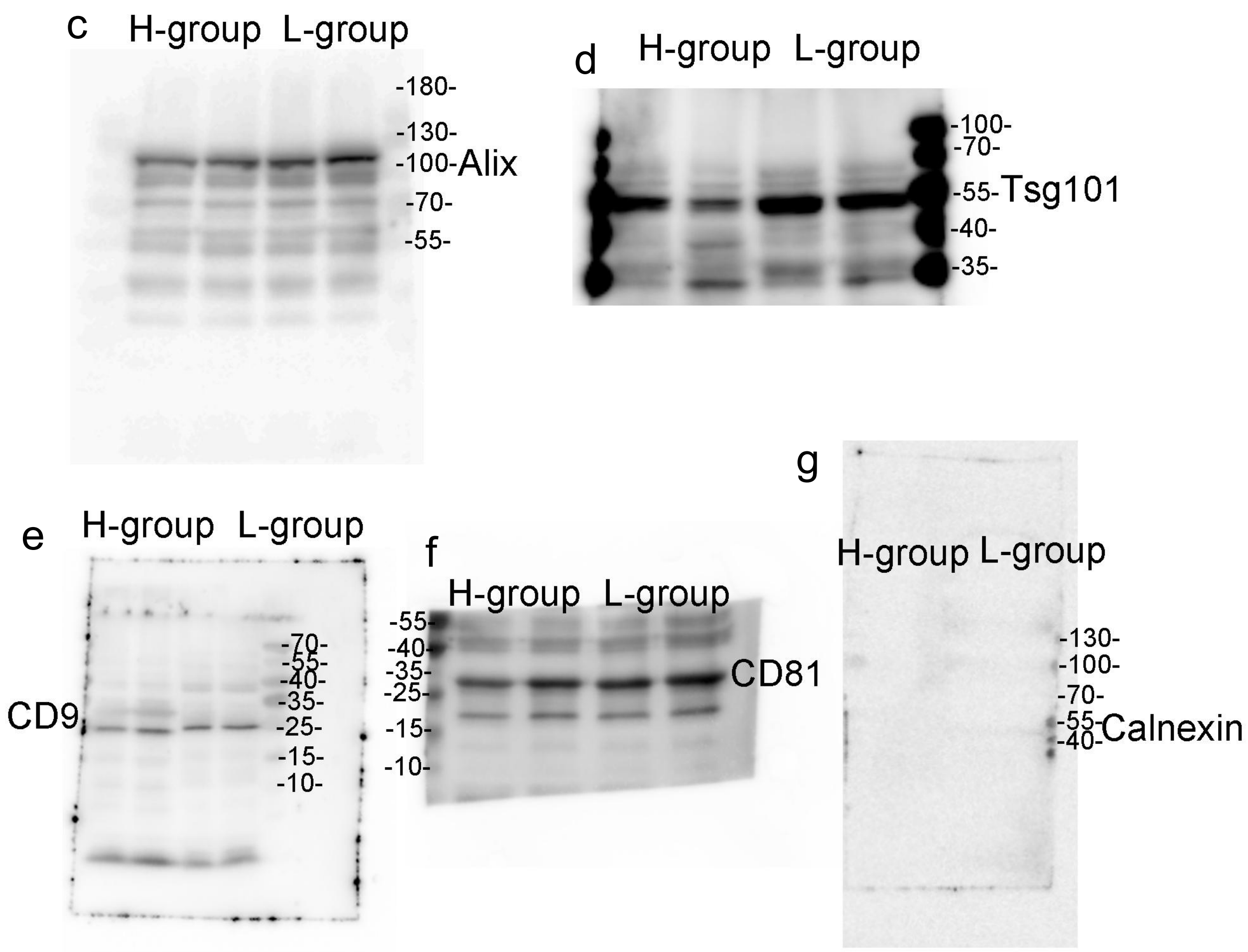

Supplementary Fig. 1: TEM and Western blotting results of SPEVs (Fig 1b, Fig 1d). TEM image of SPEVs: (**a**) H-group and (**b**) L-group. EV markers: (**c**) Alix, (**d**) Tsg101, (**e**) CD9 and (**f**) CD81. Negative EV marker: (**g**) Calnexin. H-group represents high sperm motility group and L-group represents low sperm motility group.

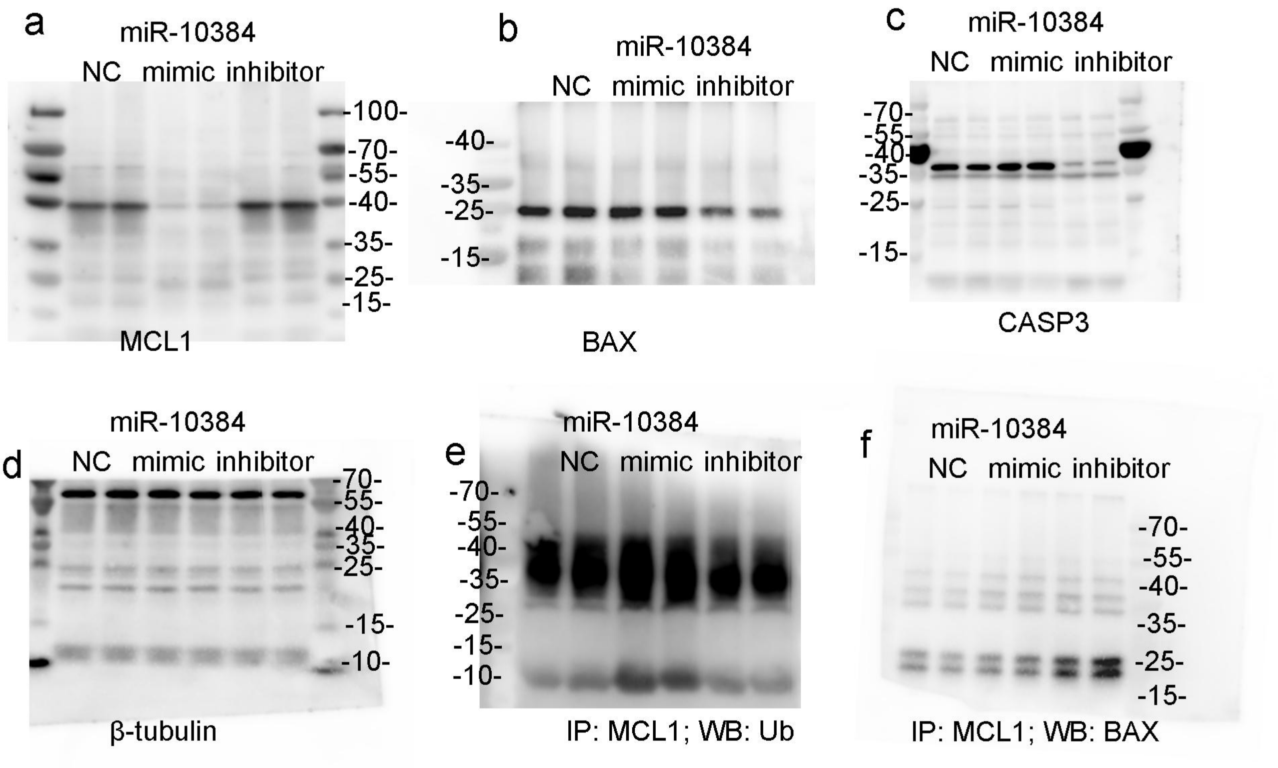

Supplementary Fig. 2: Western blot results showed that miR-10384 participates in the survival of sperm via the PI3K/Akt pathway (Fig. 7a). (a) MCL1, (**b**) BAX, (**c**) CASP3 and (**d**) β-tubulin, (**e**) the ubiquitination level of MCL1 and (**f**) the BAX levels combined with MCL1 in sperm incubated with EV (NC), EV-miR-10384 mimic, and EV-miR-10384 inhibitor.

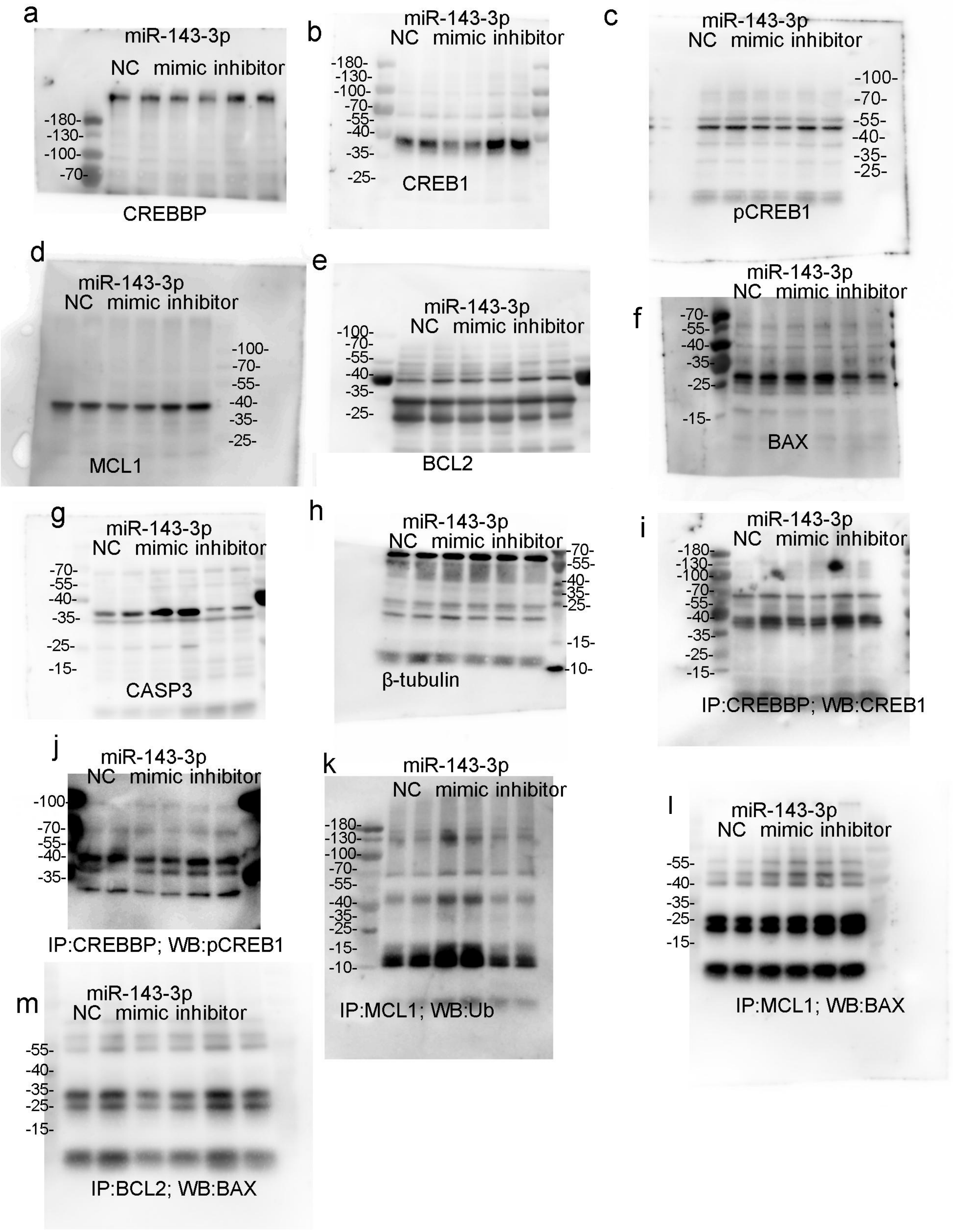

Supplementary Fig. 3: Western blot results showed that miR-143-3p participates in the survival of sperm via the PI3K/Akt pathway (Fig. 7a). (**a**) CREBBP, (**b**) CREB1, (**c**) pCREB1, (**d**) MCL1, (**e**) BCL2, (**f**) BAX, (**g**) CASP3 and (**h**) β-tubulin, (**i**) the CREB1 combined with CREBBP, (**j**) the pCREB1 combined with CREBBP, (**k**) the ubiquitination level of MCL1, (**l**) the BAX levels combined with MCL1 and (**m**) the BAX levels combined with BCL2 in sperm incubated with EV (NC), EV-miR-143-3p mimic, and EV-miR-143-3p inhibitor.

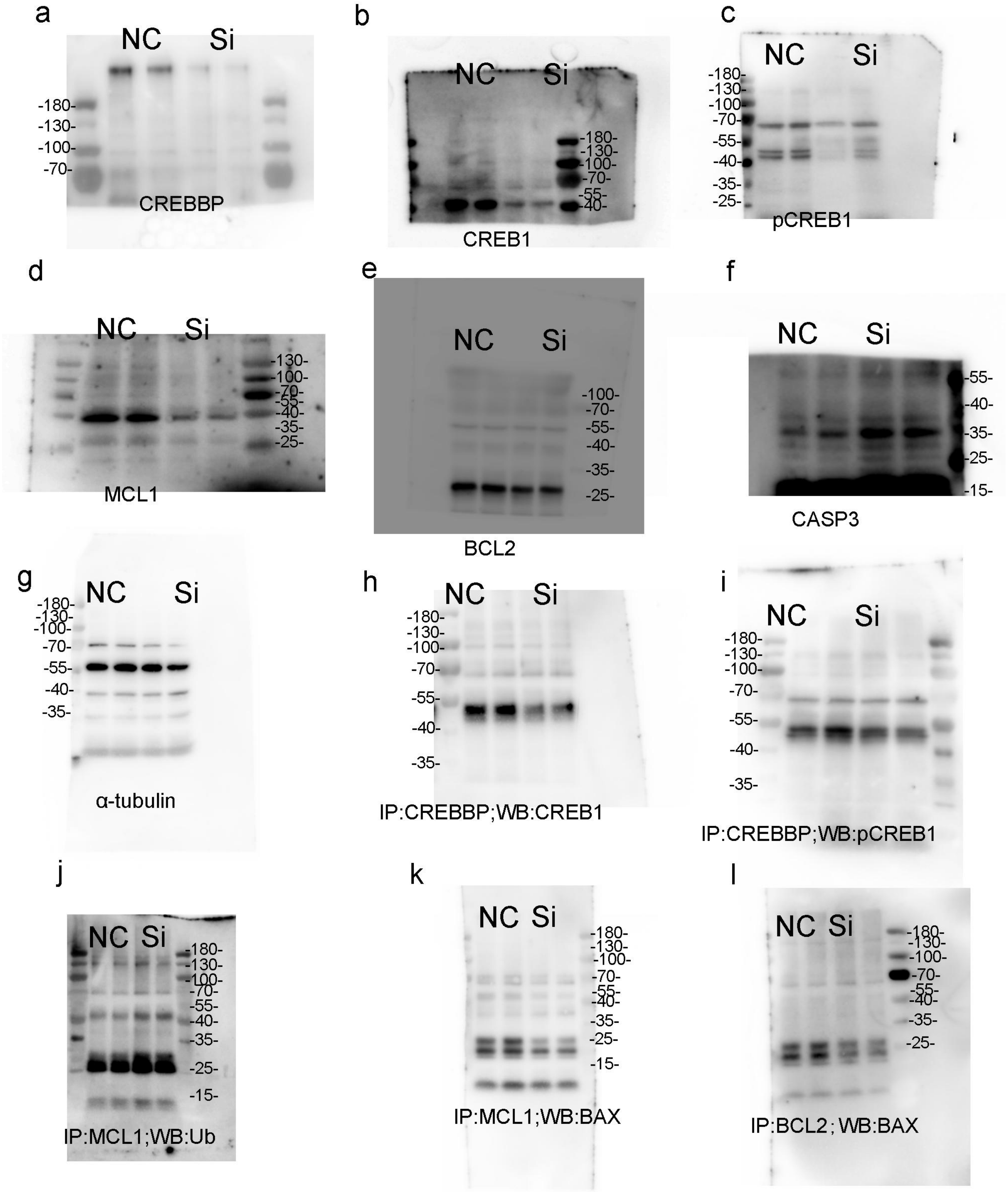

Supplementary Fig. 4: Western blot assays showed that circ-CREBBP participates in the survival of sperm via the PI3K/Akt pathway (Fig. 7d). (**a**) CREBBP, (**b**) CREB1, (**c**) pCREB1, (**d**) MCL1, (**e**) BCL2, (**f**) CASP3 and (**g**) α-tubulin, (**h**) the CREB1 combined with CREBBP, (**i**) the pCREB1 combined with CREBBP, (**j**) the ubiquitination level of MCL1, (**k**) the BAX levels combined with MCL1 and (**l**) the BAX levels combined with BCL2 in sperm incubated with EV (NC) and EV-Si-circCREBBP.

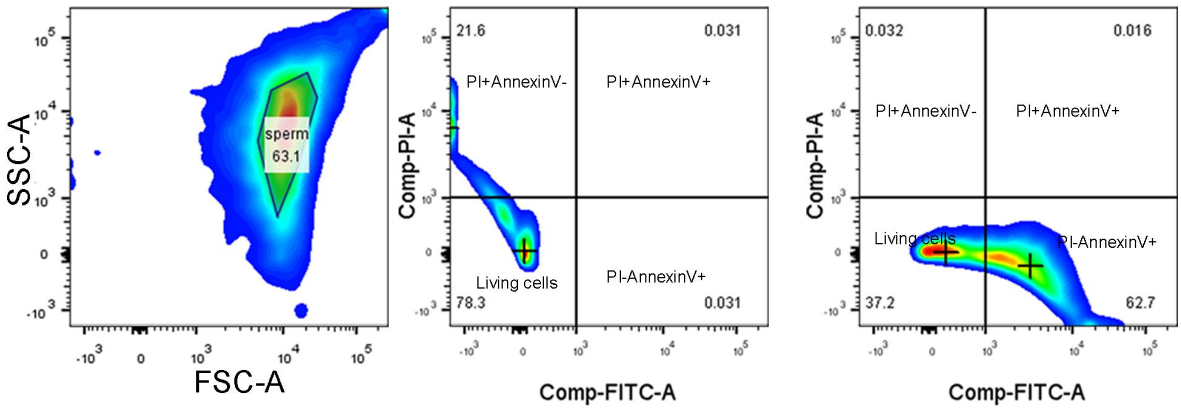

Supplementary Fig. 5: Flow cytometry gating strategy. For cell survival rate analysis, a cell gate was selected in a SSC-A versus FSC-A plot. Cells were further plotted as PI versus AnnexinV-FITC to allow identification of necrosis or apoptosis cells.

Supplementary Table 1 Host genes harboring 20 or more circRNA isoforms.

Gene name	Ensembl ID	Number of circRNA
TRIM24	ENSSSCG00000016518	20
CCDC18	ENSSSCG00000006897	20
CCDC30	ENSSSCG00000003965	20
COG3	ENSSSCG00000023738	21
SLC5A10	ENSSSCG00000018049	21
ULK4	ENSSSCG00000025408	21
KMT2C	ENSSSCG00000020663	21
USP47	ENSSSCG00000013402	21
DTNB	ENSSSCG00000008576	22
DCAF6	ENSSSCG00000006303	22
-	ENSSSCG00000011117	23
UBR3	ENSSSCG00000015940	23
UBAP2	ENSSSCG00000010987	24
NEK1	ENSSSCG00000009714	24
SEC24A	ENSSSCG00000014304	25
ATAD2B	ENSSSCG00000008591	25
IQGAP2	ENSSSCG00000014088	27
BIRC6	ENSSSCG00000008513	28
PATJ	ENSSSCG00000039793	28
MYO9A	ENSSSCG00000004979	30
ABCB5	ENSSSCG00000015377	30
BRWD1	ENSSSCG00000021042	34
CHD2	ENSSSCG00000002266	40
SMARCC1	ENSSSCG00000011340	44

Supplementary Table 2 Significant GO terms for host genes of DECs.

Term	ID	Input number	Corrected P-Value	Input
cytosol	GO:0005829	44	1.52E-08	PNPT1|FAM120A|SIRT1|AFTPH|USP9X|ADAM17|AACS|EED|PRPF3|ANKRD12|RB1CC1|RPTOR|CDK19|MAP3K2|UBXN2A|NFKB1|MVP|ARFIP1|GSPT1|SPAG9|MTR|PKN2|CHM|USP12|ILKAP|KLHL3|TP53BP2|PHLDB2|EP300|YAP1|PEX1|STX8|LRBA|PPP2CA|AKR1D1|SFMBT2|TIA1|PLEKHA1|TTI2|PPP6R2|RNF216|ATXN2|TUBGCP5|ARHGAP18
cytoplasm	GO:0005737	42	9.24E-06	TSN|KLHL24|MYO3B|SVIL|GNPDA1|MX2|ARHGAP18|FKBP5|SBF2|ITIH4|PPP4R3B|RPTOR|EPS15|UBAP2L|SAE1|GNB4|REPS2|MARK3|MAPK9|MVP|ARFIP1|SIRT1|MACF1|ARIH2|SPAG9|TRIM24|ARPC2|MDFIC|CHM|UBR3|PHLPP1|YAP1|SOS1|LIMA1|PTPN11|ROCK1|TTC17|STK33|PLEKHA1|TCF12|CCNI|CREBBP
chromatin binding	GO:0003682	12	8.42E-04	TRIM24|WAC|SMC3|STAG1|PHF21A|TOP1|SFMBT2|EED|NFKB1|ATAD2B|CREBBP|YAP1
nucleus	GO:0005634	40	8.42E-04	TSN|HMGXB3|FAM120A|PSPC1|USP9X|CCAR1|POLK|CDK19|SLC11A2|UBAP2L|CHD9|CAPN7|UBXN2A|ZC3H3|MAPK9|ZFC3H1|MVP|SIRT1|MX2|MDFIC|STAG1|CHM|USP12|AGAP1|ILKAP|TP53BP2|TCF12|GPATCH1|SBNO1|PPP2CA|YAP1|PTPN11|SFMBT2|CCNI|TFCP2|STK33|ACAD9|PPP6R2|ATAD2B|KDM5A
actin cytoskeleton	GO:0015629	8	1.82E-03	MYO3B|SVIL|LIMA1|MACF1|FILIP1|TTC17|ABLIM1|ADAM17
cellular response to UV	GO:0034644	4	0.01	EP300|POLK|CREBBP|RHBDD1
transcription coactivator activity	GO:0003713	7	0.01	SIRT1|TRIM24|MED6|EP300|KDM5A|CREBBP|YAP1
protein C-terminus binding	GO:0008022	6	0.01	PEX1|SIRT1|PPP2CA|EP300|ATXN2|YAP1
cytoplasmic stress granule	GO:0010494	4	0.02	RPTOR|ATXN2|ROCK1|TIA1
sister chromatid cohesion	GO:0007062	3	0.02	ESCO1|STAG1|SMC3

chromatin DNA binding	GO:0031490	4	0.02	SBNO1|EP300|KDM5A|CREBBP
ATP binding	GO:0005524	20	0.02	PEX1|CDK19|MYO3B|EARS2|KIF1B|CHD9|SMC3|PKN2|EIF2AK4|TOP1|ROCK1|MAP3K2|DGKH|STK33|RPS6KC1|MARK3|AACS|ATAD2B|MAPK9|CCT6B
transcription corepressor activity	GO:0003714	6	0.02	HDAC9|SFMBT2|RCOR1|CCAR1|CREBBP|YAP1
p53 binding	GO:0002039	4	0.02	TRIM24|EP300|CREBBP|TP53BP2
zinc ion binding	GO:0008270	13	0.02	MORC3|EP300|EARS2|ZMYM4|ZMYM5|TRIM24|ESCO1|MTR|SEC24A|UBR3|KDM2B|KDM5A|CREBBP
meiotic cell cycle	GO:0051321	3	0.03	PPP2CA|SMC3|TUBGCP5
positive regulation of G1/S transition of mitotic cell cycle	GO:1900087	3	0.03	RPTOR|KMT2E|ADAM17
face morphogenesis	GO:0060325	3	0.04	PTPN11|EP300|PLEKHA1
response to hypoxia	GO:0001666	4	0.04	PLOD2|EP300|SLC11A2|ADAM17
negative regulation of protein kinase B signaling	GO:0051898	3	0.05	SIRT1|PHLPP1|PLEKHA1
regulation of cell motility	GO:2000145	2	0.05	PKN2|ARHGAP18
actin filament binding	GO:0051015	6	0.05	MYO3B|SVIL|ARPC2|MACF1|ABLIM1|LIMA1
epidermal growth factor receptor signaling pathway	GO:0007173	3	0.05	PTPN11|SOS1|ADAM17
histone binding	GO:0042393	5	0.05	SIRT1|SBNO1|SUPT6H|SFMBT2|ATAD2B
multicellular organism growth	GO:0035264	4	0.05	PTPN11|EP300|PLEKHA1|SOS1

Supplementary Table 3 Phenotypic information of experimental animals.

Group	Age	Sperm motility rate (%)
H	16	90.86
	18	93.73
	17	85.09
	31	93.31
	21	88.82
	19	90.16
	14	98.50
	15	98.70
	29	99.60
	35	96.80
	30	97.70
	27	99.60
mean ± SD	22.67±6.97	94.41±4.82
L	31	59.46
	15	20.67
	22	13.59
	24	47.34
	14	13.06
	22	28.10
	31	10.08
	14	63.20
	36	73.20
	17	65.80
	29	55.30
	28	31.30
mean ± SD	23.58±7.18	40.09±23.12
P-value	0.76	6.35E-08

Supplementary Table 4 The primer sequences for qPCR.

Primer name	Sequence (5'-3')
circ-CREBBP-F	CTGTCCTGTTTGCCTCCCTT
circ-CREBBP-R	CCTCTGACACTTGTGAGCGT
circ-KLHL3-F	GTCTCAGTTGCATCCCACCA
circ-KLHL3-R	TAACCTTGAACGCCTTCCCC
circ-EP300-F	TTCCTCAGGCTCAGTTCCCA
circ-EP300-R	AAGCAGCAGGATCCGGAGTA
circ-SLC5A10-F	CGACGTGGGAGGAAGGATTG
circ-SLC5A10-R	CGACAACCTCCTTTTAGTCTCTGAC
circ-PTGES3-F	CCAAGCATAAAAGAACGGACAG
circ-PTGES3-R	CCCTTCGATCGTACCACTTT
circ-SUGCT-F	CCTTGTCTACTGCTCCATCACA
circ-SUGCT-R	CCCAAGTTCGTGTGTCATCA
miR-10384-RT	GTCGTATCCAGTGCAGGGTCCGAGGTATTCGCACTGGATACGACTGCACA
miR-10384-F	GACCCTGCGTGGCTTCTC
miR-10384-R	GTGCAGGGTCCGAGGT
miR-143-3p-RT	GTCGTATCCAGTGCAGGGTCCGAGGTATTCGCACTGGATACGACTGAGCTA
miR-143-3p-F	GCCCTGAGATGAAGCACTG
miR-143-3p-R	GTGCAGGGTCCGAGGT
U6-RT	AACGCTTCACGAATTTGCGT
U6-F	CTCGCTTCGGCAGCACA
U6-R	AACGCTTCACGAATTTGCGT
MCL1-F	TTGGGAGGCCTTGAAGAGTA
MCL1-R	TGAAGTCCGTGGGCATATTT
CREBBP-F	TGACTGTCCTGTTTGCCTCC
CREBBP-R	GAGTCCAAGAGCCGCATAGG
CREB1-F	CAGACATACCAAATTCGCACAG
CREB1-R	ATTAGGCGGACCTCTCTCTTTC
GAPDH-F	TGGTGAAGGTCGGAGTGAAC
GAPDH-R	GGAAGATGGTGATGGGATTTC
ACTB-F	GGCATCCTGACCCTCAAGTA
ACTB-R	CACGCAGCTCGTTGTAGAAG

Supplementary Table 5 Primers used in luciferase reporter assays.

Primer name	Sequence (5'-3')
pmirGLO-F	GGTGGTGTTGTGTTCGTG
pmirGLO-R	GCATCAATGTATCTTATCA
circ-CREBBP-WT-F	CTAGTTGTTTAAACGCTGGGAAAGCCTGTCAGGTT
circ-CREBBP-WT-R	CAGGTCGACTCTAGTTTGCGCTTCTCTGGGTCTG
circ-CREBBP-MUT1-F1	GACGAGGTGCCTAAAGGACTGA
circ-CREBBP-MUT1-R1	CCCGTGGCAATCGTTACTCTG
circ-CREBBP-MUT1-F2	GAATTGTGCCCAGAGTAACGATT
circ-CREBBP-MUT1-R2	TCCAAACTCATCAATGTATCTTATCA
circ-CREBBP-MUT2-F1	GACGAGGTGCCTAAAGGACTGA
circ-CREBBP-MUT2-R1	CAGTTCTTCCAATGAAGGCTACATTT
circ-CREBBP-MUT2-F2	ATCTTCACGACAAATGTAGCCTCAT
circ-CREBBP-MUT2-R2	TCCAAACTCATCAATGTATCTTATCA
MCL1-WT-F	CTAGTTGTTTAAACGGACGAGGTGCCTAAAGGACT
MCL1-WT-R	CAGGTCGACTCTAGTCCAAACTCATCAATGTATC
MCL1-MUT-F	TTCTGACTCATAATTCTAACTGCTTTAATTTT
MCL1-MUT-R	AAAATTAAAGCAGTTAGAATTATGAGTCAG AA
CREB1-WT-F	CTAGTTGTTTAAACGAGTATTTGGTTTCTATGCTTTGTCTC
CREB1-WT-R	CAGGTCGACTCTAGCAGAGATAAACTTCAGCCAGATGAC
CREB1-MUT-F1	CTAGTTGTTTAAACGAGTATTTGGTTTCTAGTAGGAAGAGATGGCA
CREB1-MUT-F2	TAGTAGGAAGAGATGGCAGCTATAACAATGGAAA
CREB1-MUT-R	CAGGTCGACTCTAGCATCTCGCCACTTCAGCCAGATGAC
CREBBP-WT-F	CTAGTTGTTTAAACGGCGTCTCCCAGTATTACCCCT
CREBBP-WT-R	CAGGTCGACTCTAGTCCTCATTTCAAGTTTCACATAGA
CREBBP-MUT-F	ATGTAGAAATTGTACGAATGATAGTCTGATAA
CREBBP-MUT-R	TTATCAGACTATCATTCGTACAATTTCTACAT
