# Supplementary material for: Circ-CREBBP inhibits sperm apoptosis via the PI3K-Akt signaling pathway by sponging miR-10384 and miR-143-3p
Source: Commun Biol. 2022 Dec 7;5:1339. doi: 10.1038/s42003-022-04263-2 (PMC9729231; doi:10.1038/s42003-022-04263-2)
Supplement: Supplementary file 3 — Description of Additional Supplementary Files [file 42003_2022_4263_MOESM3_ESM.docx]

**Description of Additional Supplementary Files**

**File name:** Supplementary Data 1

**Description:** Highly conserved circRNAs between pigs and humans. There is no corresponding figure in the paper.

**File name:** Supplementary Data 2

**Description:** The source data behind the Figure 2g in the paper.

**File name:** Supplementary Data 3

**Description:** The source data behind the Figure 3a in the paper.

**File name:** Supplementary Data 4

**Description:** The source data behind the Figure 4a,b in the paper.

**File name:** Supplementary Data 5

**Description:** The source data behind the graphs in Figure in the paper.
