# Supplementary material for: Circ-CREBBP inhibits sperm apoptosis via the PI3K-Akt signaling pathway by sponging miR-10384 and miR-143-3p
Source: Commun Biol. 2022 Dec 7;5:1339. doi: 10.1038/s42003-022-04263-2 (PMC9729231; doi:10.1038/s42003-022-04263-2)
Supplement: Supplementary file 9 — Reporting Summary [file 42003_2022_4263_MOESM9_ESM.pdf]

nature portfolio

Corresponding author(s): Li Jiang

Last updated by author(s): Nov 3, 2022

Reporting Summary

Nature Portfolio wishes to improve the reproducibility of the work that we publish. This form provides structure for consistency and transparency in reporting. For further information on Nature Portfolio policies, see our Editorial Policies and the Editorial Policy Checklist.

Statistics

For all statistical analyses, confirm that the following items are present in the figure legend, table legend, main text, or Methods section.

n/a	Confirmed	
☐	☒	The exact sample size (*n*) for each experimental group/condition, given as a discrete number and unit of measurement
☐	☒	A statement on whether measurements were taken from distinct samples or whether the same sample was measured repeatedly
☐	☒	The statistical test(s) used AND whether they are one- or two-sided<br>*Only common tests should be described solely by name; describe more complex techniques in the Methods section.*
☒	☐	A description of all covariates tested
☐	☒	A description of any assumptions or corrections, such as tests of normality and adjustment for multiple comparisons
☐	☒	A full description of the statistical parameters including central tendency (e.g. means) or other basic estimates (e.g. regression coefficient) AND variation (e.g. standard deviation) or associated estimates of uncertainty (e.g. confidence intervals)
☐	☒	For null hypothesis testing, the test statistic (e.g. *F*, *t*, *r*) with confidence intervals, effect sizes, degrees of freedom and *P* value noted<br>*Give P values as exact values whenever suitable.*
☒	☐	For Bayesian analysis, information on the choice of priors and Markov chain Monte Carlo settings
☒	☐	For hierarchical and complex designs, identification of the appropriate level for tests and full reporting of outcomes
☒	☐	Estimates of effect sizes (e.g. Cohen's *d*, Pearson's *r*), indicating how they were calculated

Our web collection on statistics for biologists contains articles on many of the points above.

Software and code

Policy information about availability of computer code

Data collection	No code was used to collect data in this study - code was only used for data analysis
Data analysis	bwa (v 0.7.17-r1188) (https://github.com/lh3/bwa)<br>samtools (v 1.10) (https://github.com/samtools)<br>bowtie (v 1.0.0) (http://bowtie-bio.sourceforge.net/index.shtml)<br>bowtie2 (v 2.4.5) (http://bowtie-bio.sourceforge.net/bowtie2/index.shtml)<br>CIRI (v 2.0.6) (https://sourceforge.net/projects/ciri/files/CIRI2/CIRI_v2.0.6.zip/download)<br>find_circ (https://github.com/marvin-jens/find_circ)<br>cutadapt (v 3.5) (https://github.com/marcelm/cutadapt/)<br>miRDeep2.0.1.2 (https://github.com/rajewsky-lab/mirdeep2)<br>miranda v3.3a (http://cbio.mskcc.org/microrna_data/miRanda-aug2010.tar.gz)<br>RNAhybrid (https://bibiserv.cebitec.uni-bielefeld.de/rnahybrid)<br>Cytoscape (v 3.8.0) (https://cytoscape.org/)<br>Kobas (http://kobas.cbi.pku.edu.cn/)<br>liftOver (https://genome.ucsc.edu/cgi-bin/hgLiftOver)<br>blastn (http://blast.ncbi.nlm.nih.gov)<br>GraphPad Prism (v 8.0.1) (https://www.graphpad.com/scientific-software/prism/)<br>R (v 4.1.2) (https://www.r-project.org/)<br>ggplot2 (v 3.3.5) package<br>ggsci (v 2.9) package<br>edgeR (v 3.36.0) package

VennDiagram (v 1.7.3) package
ggrepel (v 0.9.1) package
networkD3 (0.4) package

For manuscripts utilizing custom algorithms or software that are central to the research but not yet described in published literature, software must be made available to editors and reviewers. We strongly encourage code deposition in a community repository (e.g. GitHub). See the Nature Portfolio guidelines for submitting code & software for further information.

Data

Policy information about availability of data

All manuscripts must include a data availability statement. This statement should provide the following information, where applicable:

- Accession codes, unique identifiers, or web links for publicly available datasets
- A description of any restrictions on data availability
- For clinical datasets or third party data, please ensure that the statement adheres to our policy

The datasets generated during and/or analysed during the current study are available from the corresponding author on reasonable request.

Human research participants

Policy information about studies involving human research participants and Sex and Gender in Research.

Reporting on sex and gender	N/A
Population characteristics	N/A
Recruitment	N/A
Ethics oversight	N/A

Note that full information on the approval of the study protocol must also be provided in the manuscript.

Field-specific reporting

Please select the one below that is the best fit for your research. If you are not sure, read the appropriate sections before making your selection.

☒ Life sciences ☐ Behavioural & social sciences ☐ Ecological, evolutionary & environmental sciences

For a reference copy of the document with all sections, see nature.com/documents/nr-reporting-summary-flat.pdf

Life sciences study design

All studies must disclose on these points even when the disclosure is negative.

Sample size	The datasets involved in this study was determined by specific phenotype information, such as total sperm motility and fast forward motility. All experimental individuals were from a national boar station. About 10% of individuals in the population had sperm motility lower than 0.6. We selected individuals with extremely low sperm motility in the population (5% in the lower tail) and healthy individuals (5% in the upper tail) in the the same period.
Data exclusions	No data was excluded. Samples were allocated to groups based on the sperm motility.
Replication	Experiments were with three biological replicates and three technical replicates.
Randomization	All pigs without physiological or pathological diseases were from a national boar station. Pigs were all sexually mature, aged between 14 and 36 months old. There was no significant difference between the two groups in the age of pigs. The samples are collected by the same professional staff in autumn (from October to November 10).
Blinding	The blinding was not relevant to our study.<br>Different people completed the sample collection and data analysis.

Reporting for specific materials, systems and methods

We require information from authors about some types of materials, experimental systems and methods used in many studies. Here, indicate whether each material, system or method listed is relevant to your study. If you are not sure if a list item applies to your research, read the appropriate section before selecting a response.

Materials & experimental systems

n/a	Involved in the study	
☐	☒	Antibodies
☐	☒	Eukaryotic cell lines
☒	☐	Palaeontology and archaeology
☐	☒	Animals and other organisms
☒	☐	Clinical data
☒	☐	Dual use research of concern

Methods

n/a	Involved in the study	
☒	☐	ChIP-seq
☐	☒	Flow cytometry
☒	☐	MRI-based neuroimaging

Antibodies

Antibodies used

Antibodies are listed below as: target; Host; supplier name;catalog number; clone number; lot number:
1)Alix; Mouse; Santa Cruz; Cat# sc-53540; CloneNo.1A12;
2)TSG101; Rabbit; Proteintech; Cat# 14497-1-AP; CloneNo. n/a; Lot# 00093288;
3)CD9; Rabbit; Proteintech; Cat# 20597-1-AP; CloneNo. n/a; Lot# 00055311;
4)CD81; Mouse; Proteintech; Cat# 66866-1-Ig; CloneNo. 1G2C6; Lot# 10017531;
5)Calnexin; Proteintech; Cat# 10427-2-AP;
6)MCL1; Rabbit; Abmart; Cat# T55199S; CloneNo. n/a; Lot# 334280;
7)CREBBP; Rabbit; Abmart; Cat# PA1273S; CloneNo. n/a; Lot# 334362;
8)CREB1; Mouse;Abmart; Cat# MB0153S; CloneNo. n/a; Lot# 334261;
9)p-CREB1; Mouse; Santa Cruz; Cat# sc-81486; CloneNo. 1019; Lot#E1721 ;
10)BCL2; Rabbit; Proteintech; Cat# 12789-1-AP; CloneNo. n/a; Lot# 00095186;
11)BAX; Mouse; Proteintech; Cat# 60267-1-Ig; CloneNo. 4G5E8; Lot# 10005017;
12)Caspase-3; Rabbit; Abcam; Cat# ab13847; CloneNo. n/a; Lot# GR3286166-1;
13)β-Tubblin; Rabbit; Proteintech; Cat# 10068-1-AP,CloneNo. n/a; Lot# 00085542;
14)α-Tubblin; Rabbit; Proteintech; Cat# 11224-1-APCloneNo. n/a; Lot# 00093821;

Validation

All antibody specificity was validated by the manufacture, and the validation result is posted on their website.

Eukaryotic cell lines

Policy information about cell lines and Sex and Gender in Research

Cell line source(s)	293T [HEK-293T] is the preservative cell line in the Lab.
Authentication	cell lines were not validated.
Mycoplasma contamination	All cell lines tested negative for mycoplasma.
Commonly misidentified lines (See ICLAC register)	N/A

Animals and other research organisms

Policy information about studies involving animals; ARRIVE guidelines recommended for reporting animal research, and Sex and Gender in Research

Laboratory animals	N/A
Wild animals	N/A
Reporting on sex	N/A
Field-collected samples	All pigs are kept under the same conditions and environment (e.g. field, temperature, food etc.). Specialized professionals obtained the sperm-rich fractions of the ejaculate sample from each boar by the gloved hand method. All samples were quickly transferred to the laboratory at 17 ℃.
Ethics oversight	All protocols for the collection of semen samples were reviewed and approved by the Committees for Ethical Review of China Agricultural University.

Note that full information on the approval of the study protocol must also be provided in the manuscript.

Flow Cytometry

Plots

Confirm that:

☒ The axis labels state the marker and fluorochrome used (e.g. CD4-FITC).

☒ The axis scales are clearly visible. Include numbers along axes only for bottom left plot of group (a 'group' is an analysis of identical markers).

☐ All plots are contour plots with outliers or pseudocolor plots.

☒ A numerical value for number of cells or percentage (with statistics) is provided.

Methodology

Sample preparation	SPEVs were electroporated with Si-circRNA (sense: 5'- GCGAA ACCAA CAAAU CUCAT T -3'; antisense: 5'- UGAGA UUUGU UGGUU UCGCT T -3') using a Gene Pulser Xcell electroporator (Bio–Rad, USA) with the "exponential" protocol (250 V, 100 μF, R=∞, cuvette size=4 mm) .<br>The sperm were separated from semen samples by centrifuging at 800 × g for 10 minutes at 17 ℃, and coincubated with electrotransfected SPEVs at 17 ℃. Sperm cells were collected on days 2 and 4 for evaluation of cell apoptosis.<br>The sperm apoptotic rate was evaluated according to the protocols provided by the Annexin V-FITC Apoptosis Detection Kit (Beyotime, China). Generally, 1×10^5 sperm cells were diluted within buffer, and added with Annexin V-FITC and PI. The cell mixture was cultured at room temperature for 20 minutes and then analyzed by a BD LSRFortessa flow cytometer.
Instrument	BD LSRFortessa flow cytometer
Software	Flow Jo V10
Cell population abundance	For analysis results, the plot result is divided into four quadrants , Q1 (PI-A+, FITC-A-) mainly contained necrotic cells, Q2 (PI-A +, FITC-A+) mainly contained apoptosis cells, Q3 (PI-A-, FITC-A+) mainly contained early apoptosis cells, Q4 (PI-A-, FITC-A-) mainly contained surviving cells.
Gating strategy	Cell clusters were first identified by physical parameters: the FSC-A and the SSC-A.<br>Classfication of positive and negative populations were done through Staining Protocols (Apoptosis) and Eliminating Dead Cells from Analysis.

☒ Tick this box to confirm that a figure exemplifying the gating strategy is provided in the Supplementary Information.
